# Automated Opportunistic Osteoporosis Screening Using Low-Dose Chest CT among Individuals Undergoing Lung Cancer Screening in a Korean Population

**DOI:** 10.3390/diagnostics14161789

**Published:** 2024-08-16

**Authors:** Woo Young Kang, Zepa Yang, Heejun Park, Jemyoung Lee, Suk-Joo Hong, Euddeum Shim, Ok Hee Woo

**Affiliations:** 1 Department of Radiology, Korea University Guro Hospital, Seoul 08308, Republic of Korea; quartet0@hanmail.net (W.Y.K.); yangzepa@gmail.com (Z.Y.); eirbadmin@kumc.or.kr (H.P.); hongsj@korea.ac.kr (S.-J.H.); 2 Department of Applied Bioengineering, Seoul National University, Seoul 08826, Republic of Korea; jaymlee0407@snu.ac.kr; 3 ClariPi Research, ClariPi Inc., Seoul 03088, Republic of Korea; 4 Department of Radiology, Korea University Ansan Hospital, Ansan 15355, Republic of Korea; edhim1214@gmail.com

**Keywords:** osteoporosis, opportunistic screening, computed tomography, bone mineral density, deep learning, prevalence

## Abstract

Opportunistic osteoporosis screening using deep learning (DL) analysis of low-dose chest CT (LDCT) scans is a potentially promising approach for the early diagnosis of this condition. We explored bone mineral density (BMD) profiles across all adult ages and prevalence of osteoporosis using LDCT with DL in a Korean population. This retrospective study included 1915 participants from two hospitals who underwent LDCT during general health checkups between 2018 and 2021. Trabecular volumetric BMD of L1-2 was automatically calculated using DL and categorized according to the American College of Radiology quantitative computed tomography diagnostic criteria. BMD decreased with age in both men and women. Women had a higher peak BMD in their twenties, but lower BMD than men after 50. Among adults aged 50 and older, the prevalence of osteoporosis and osteopenia was 26.3% and 42.0%, respectively. Osteoporosis prevalence was 18.0% in men and 34.9% in women, increasing with age. Compared to previous data obtained using dual-energy X-ray absorptiometry, the prevalence of osteoporosis, particularly in men, was more than double. The automated opportunistic BMD measurements using LDCT can effectively predict osteoporosis for opportunistic screening and identify high-risk patients. Patients undergoing lung cancer screening may especially profit from this procedure requiring no additional imaging or radiation exposure.

## 1. Introduction

Osteoporosis is a systemic skeletal disorder characterized by low bone mass and microarchitectural deterioration of bone tissue, resulting in compromised bone strength that predisposes individuals to an elevated risk of fracture [1]. It is a highly prevalent geriatric disorder. As Korea is rapidly approaching a ‘super-aging society’, osteoporosis is emerging as a major public healthcare concern. The socioeconomic burden and mortality rates associated with osteoporosis and its related fractures have notably increased [2,3]. A cross-sectional nationwide health survey revealed that among Korean adults aged 50 years and older, the prevalence of osteoporosis and osteopenia was 22.4% and 47.9%, respectively [4]. Nevertheless, a lot of patients with osteoporosis or related fractures still remain underdiagnosed and under-treated because osteoporosis, known as “the quiet thief of the bone”, remains asymptomatic until fractures occur. Therefore, the early detection of osteoporosis is crucial for the timely prevention and treatment of osteoporotic fractures.

Bone density measurement plays an important role in the assessment of osteoporosis. Currently, dual X-ray absorptiometry (DXA) serves as the gold standard for measuring bone mineral density (BMD) for osteoporosis diagnosis [5]. However, DXA is underutilized as it is a two-dimensional technique, which can result in inaccuracies in patients with degenerative changes, compressive fractures, or aortic calcification [6,7,8]. Quantitative computed tomography (QCT) is another clinical standard for measuring volumetric BMD (vBMD) using three-dimensional information, but its widespread application is hindered by high radiation exposure, the need for dedicated software and phantoms, and intricate post-processing processes [9,10]. These challenges raise great interest in using alternative imaging modalities.

Opportunistic osteoporosis screening using clinical routine CT has gained attention over the past decade. This method allows for osteoporosis screening without additional time, costs, or radiation exposure, regardless of the clinical indication for imaging [11,12,13,14,15]. Recently, deep learning (DL) has emerged as a promising tool in medical imaging. Numerous studies have demonstrated that DL techniques, especially convolutional neural networks (CNNs), can automatically predict BMD and classify osteoporosis when performing opportunistic osteoporosis screening using low-dose chest computed tomography (LDCT) [9,16,17,18]. We previously developed a DL model for automated trabecular BMD measurement using QCT results as the reference standard and demonstrated that our DL model could measure the BMD of the lumbar spine from various routine CT scans with high diagnostic performance [19]. Our proposed method, utilizing DL to analyze LDCT scans, offers a promising alternative by providing automated, opportunistic volumetric BMD assessments without additional procedures or cost. This approach enhances sensitivity and accessibility, addressing the limitations of traditional methods such as DXA or QCT, and potentially improving the early detection and management of osteoporosis.

In this study, we aimed to assess the prevalence of osteoporosis by using automated BMD measurements of the lumbar spine obtained from LDCT with DL in a clinical routine setting. Previous research has demonstrated the strong diagnostic performance of our DL model, with a high correlation to QCT values, underscoring its potential as a trustworthy tool [19]. Additionally, we focused on evaluating the utility of CT-based BMD measurements for opportunistic osteoporosis screening in specific patient populations, particularly those undergoing routine LDCT scans.

## 2. Materials and Methods

This retrospective study was conducted at two academic medical centers according to the guidelines of the Declaration of Helsinki and approved by the Institutional Review Boards of the respective institutes. The requirement for informed consent was waived because of the retrospective nature of the study and the use of anonymized data.

### 2.1. Participants

**Figure 1:**
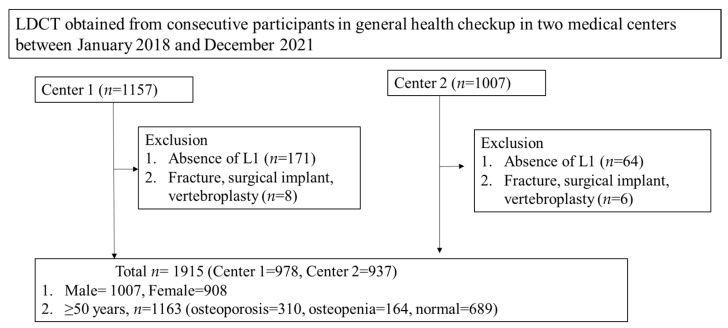
Flowchart of participants.

Between January 2018 and December 2021, 2164 participants aged 20 years or older who had undergone LDCT for lung cancer screening as part of a general health checkup at one of the two participating institutions were included retrospectively. We collected data from two different institutes to mitigate potential errors in data results arising from the clinical care protocols of a single medical institution and regional biases. Our main goal was to obtain a large, representative sample that included a broad range of patient ages. We aimed to collect 1000 cases from two hospitals, dividing the patients’ ages into 5-year clusters, with 100 male cases and 100 female cases in each cluster. Additionally, acquisition conditions were expanded to include images from outpatients, alongside low-dose CT scans from health checkups, to address shortages in specific age groups over 85. These individuals were generally healthy subjects who exhibited no discernible non-spinal disease. Among those subjects, 249 patients were excluded for the following reasons: (1) L1 vertebra not included in the CT scan (*n* = 235) or (2) trabecular BMD measurement not feasible due to fracture, surgical implant, or vertebroplasty (*n* = 14). Finally, 1915 participants (male = 1007, female = 908, mean age = 57.034 years, range 26–89 years) were enrolled in this study (Figure 1).

### 2.2. LDCT Image Acquisition

All participants underwent LDCT scanning using a variety of multidetector CT scanners from four different vendors (Aquilion One, Canon Medical Systems; Revolution CT, GE Healthcare; Brilliance 64, Ingenuity Core 128, IQon, Philips Healthcare; Sensation 16, Somatom Definition AS+, Somatom Definition Edge, Somatom Force, Siemens Healthineers). CT examinations were performed using a tube voltage of 100–135 kVp, a slice thickness of 0.668–10 mm, and variable adaptive tube current values without intravenous or oral contrast agent (Table 1). The effective dose for LDCT typically ranges between 0.3 and 1.7 millisieverts (mSv), substantially lower than the doses associated with standard CT protocols, which often range from 3.6 to 5.3 mSv.

### 2.3. Automatic vBMD Measurement

**Figure 2:**
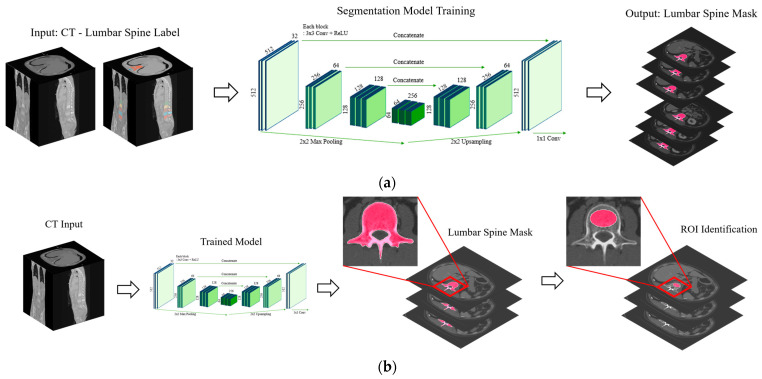
Overview of automatic volumetric bone mineral density (vBMD) measurement using deep learning comprising lumbar spine segmentation and region of interest (ROI) identification at L1 and L2. (**a**) The spine segmentation model was trained based on a U-Net architecture using only CT images paired with lumbar spine-labeled masks. We only trained the lumbar spine mask to obtain the binary mask in the lumbar range, which was used for detecting the L1 spine location to designate the vBMD ROI. (**b**) Using the lumbar spine segmentation-trained model, the first binary mask object of the output would be L1, which was the optimal location for measuring bone density. To define the specific ROI for vBMD, only the trabecular area of the spine should be covered, avoiding the basivertebral vein area. We used a morphological erosion algorithm for the elliptical ROI based on the segmented spine mask.

vBMDs were derived from LDCT images in the L1–2 vertebrae by using a fully automated tool that was previously developed and evaluated [19]. The deep learning model for automatic BMD measurement (ClariQCT Ver 1.0, ClariPi Inc., Seoul, Republic of Korea) consisted of two phases: lumbar spine segmentation and region of interest (ROI) identification at L1 and L2. We used open dataset training data and applied preprocessing techniques, including CT denoising (ClariCT.AI, ClariPi Inc., Seoul, Republic of Korea) and kernel normalization with various filters (Figure 2).

#### 2.3.1. Training and Validation Datasets

This study utilized 324 chest and abdominal CT scans from the VerSe2020 public dataset, consisting of 114,048 axial reconstructed CT images, each with corresponding binary spine masks [20]. For lumbar spine segmentation, these images were divided into training and validation datasets, with the training set comprising 70% of the images (79,834 slices) and the validation set comprising the remaining 30% (34,214 slices). These scans were acquired from various CT scanners, including the Philips Brilliance 64, iCT, and IQon, as well as the Siemens SOMATOM Definition AS and AS+. Imaging parameters included an exposure of 120 kVp, slice thickness ranging from 0.9 mm to 2.0 mm, and pixel spacing varying from 0.81 × 0.81 mm to 0.88 × 0.88 mm. Cases were categorized based on DXA results into three groups: normal (83 cases, 25.6%), osteopenia (57 cases, 17.6%), and osteoporosis (184 cases, 56.8%).

#### 2.3.2. Model Training

For all datasets, the lumbar spine (L spine) region was originally labeled sequentially, with labels increasing incrementally (e.g., L1 = 20, L2 = 21, etc.). The thoracic vertebrae were re-labeled as 0, while the lumbar vertebrae were modified to a label of 1. A U-Net network architecture, featuring an encoder and decoder module connected via concatenated skip connections, was employed for CT image analysis, utilizing dice loss as the loss function. Preprocessing included normalization and adjustment of image values using window level 300 and window width 850 settings. This study also incorporated fundamental data augmentation techniques such as smoothness/sharpness filters, translation, and rotation. Additionally, we applied AI-powered denoising (ClariCT.AI, ClariPi Inc.) in the preprocessing step. This could help normalize the input pixel noise variation and reduce the variability from diverse protocols with different CT vendors [21,22,23]. Field-of-view (FOV) adjustments were also applied in the preprocessing step, involving resizing the scaled spinal area and corresponding CT images to 512 × 512 dimensions. These measures were implemented to promote generalized learning and ensure consistent segmentation performance across varying CT scan parameters from different manufacturers.

#### 2.3.3. ROI Identification and vBMD Assessment

The spine segmentation model generates a mask specifically for the lumbar spine, allowing us to easily identify the L1 location. Subsequently, the next spine mask generated corresponds to the L2 location. At each lumbar spine area, an elliptical ROI was placed using morphologic features of each spine mask to only encompass the inner trabecular bone region and avoid the cortical bone. When calculating the average from the distribution of HU values within the ROI, errors could occur if extraordinary high HU values are present, such as those from calcification due to degeneration. To minimize the inclusion of outlier ossification lesions, the data distribution was trimmed by removing the extreme 1% of values that could be considered outliers in the HU distribution within the ROI. After excluding these extreme values, the median (50th percentile) of the remaining data distribution was calculated and designated as the representative HU value.

We utilized the European Spine Phantom (ESP) for vBMD assessment. The ESP, an anthropomorphic phantom, serves as a calibration standard for QCT and DXA. We ac-quired CT images of the ESP asynchronously, following the same protocols used for patient CT images. From these ESP images, we calculated HU values for three distinct regions of the vertebrae within the phantom, which had bone densities of 50, 100, and 200 mg/cc, respectively. We matched the HU values with each vertebra part of the specified bone densities and employed six different regression methods to derive conversion equations. Post-regression, we derived six equations, used them to estimate the original BMD values from the HU inputs, and calculated the RMSE (root mean squared error) for each equation. If an equation’s RMSE exceeded 5.0, we recalibrated the ESP to obtain a new set of HU values. Ultimately, we selected the equation with the lowest RMSE for the HU-BMD conversion. This approach was applied across various protocols, including 80, 90, 100, 110, and 120 kVp, sharp-standard kernels, FBP, and IR reconstruction methods. Consequently, we developed multiple HU-BMD equations tailored to each protocol. When calibration was required for a different manufacturer’s CT model, the process was repeated separately for each CT machine. This value was then applied to the HU-BMD conversion formula to obtain the final vBMD value.

### 2.4. Statistical Analysis

Participants were stratified by sex and age, with age groups defined in 5-year increments, ranging from 20 to 85 years and older. Calculated vBMD values were classified as indicating osteoporosis, osteopenia, or normal according to the American College of Radiology QCT diagnostic criteria, which are BMD < 80 mg/cm^3^, 80–120 mg/cm^3^, and >120 mg/cm^3^, respectively [24]. Data were summarized descriptively. Continuous variables are reported as median and interquartile range (IQR). Categorical variables are reported as frequencies and percentages. Age- and gender-specific prevalences of osteoporosis were calculated. The age-standardized prevalence was calculated using the age distribution of the Korean population in 2020. All calculations were performed with Microsoft Excel software (version 2022, Build 14931.20764). The prevalence of osteoporosis obtained from LDCT using a DL model in the current study was compared with recent DXA-based estimates published by the Korean Society for Bone and Mineral Research (KSBMR) in 2019 [4].

## 3. Results

### 3.1. Participant Demographics

**Figure 3:**
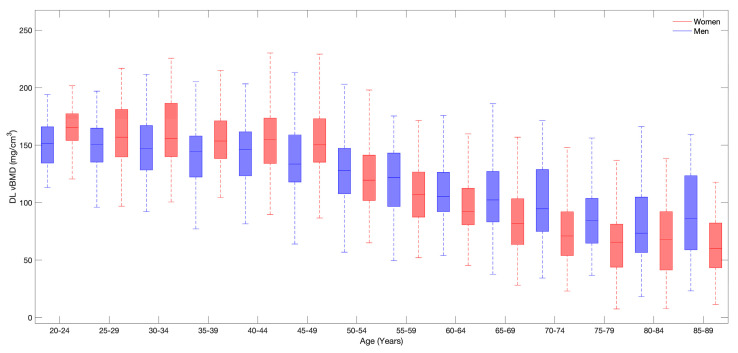
Age–related changes in deep learning-derived vertebral bone mineral density (DL vBMD, median and interquartile range) for men and women. Both sexes show a decline in DL vBMD with increasing age, with a more pronounced decrease in women from the age of 50–54 years.

The median vBMD distribution by gender and age group is summarized in Table 2. Figure 3 shows the age-stratified median and IQR of vBMD for each 5-year interval. vBMD was highest in individuals in their twenties and gradually declined with increasing age, varying in women from 165.24 to 60.07 mg/cm^3^, and in men from 151.40 to 73.36 mg/cm^3^. vBMD in men continued to decrease from the third decade of life, whereas in women, it remained nearly constant until the fourth decade, and then declined rapidly from the fifth decade. Women exhibited a higher vBMD than men before the age of 50 years, whereas older women had a lower vBMD after the age of 50 than men.

### 3.2. Prevalence of Osteoporosis

**Figure 4:**
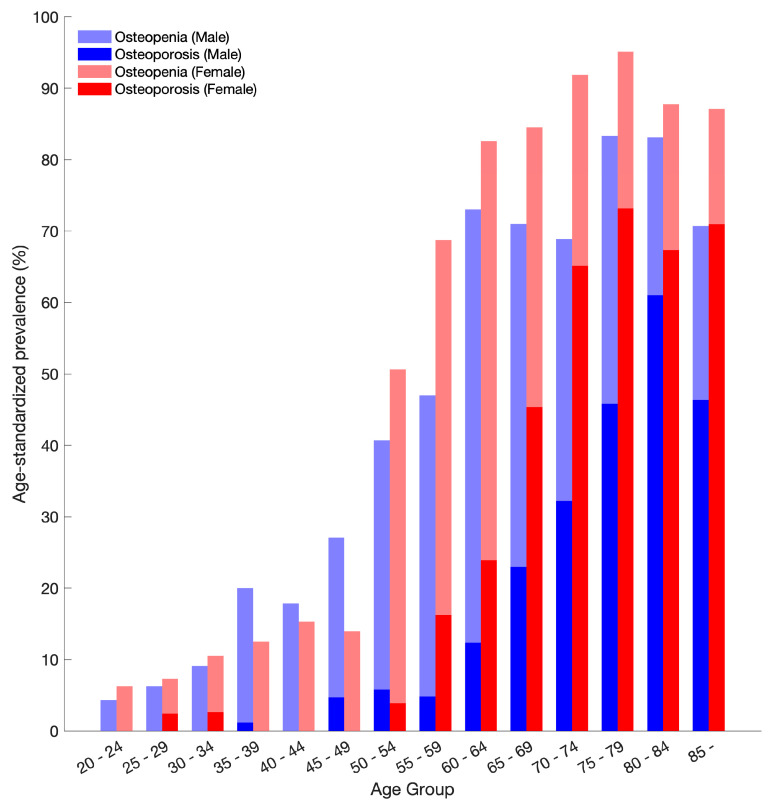
Age–standardized prevalence of osteoporosis and osteopenia stratified by age group and sex. The prevalence of osteoporosis increases with age, with a notable rise in women compared to men, particularly in the older age groups.

The prevalence of osteoporosis and osteopenia in participants is shown in Figure 4. The prevalence of both osteoporosis and osteopenia increased with age in both men and women. In men, the prevalence of osteoporosis was 5.8% at age 50 to 54 years and 61.0% at age 80 to 84 years. For women, the prevalence increased from 3.9% at age 50 to 54 years to 73.2% at age 75 to 79 years. Women in their 50s had twice the prevalence of osteoporosis as men. Women showed a higher prevalence of both conditions compared to men, particularly after the age of 55. The prevalence of osteoporosis in women surpasses that in men after this age, with a particularly steep rise in the older age groups (65–69 years and beyond). Men experience a notable increase in osteoporosis prevalence with older age groups, maintaining a consistently lower prevalence compared to women. Among men aged 70 years and older, 45.7% had osteoporosis.

Among adults aged 50 and older, 34.4% had osteoporosis and 39.0% had osteopenia. Osteoporosis affected 26.8% of men and 42.6% of women, while osteopenia was present in 39.8% of men and 38.0% of women. The previous DXA-based census reported a similar prevalence of osteopenia in men and women, but a prevalence of osteoporosis in men was only one-fifth of that in women. Compared to the DXA-based data, the prevalence of osteopenia was slightly lower in the CT-based results. In women, the age-standardized prevalence of osteoporosis was similar between the CT and DXA methods. However, in men, the age-standardized prevalence of osteoporosis detected by CT was 2.4 times higher than that reported by DXA (Table 3).

## 4. Discussion

Our study demonstrated the clinical utility and feasibility of applying an automatic DL model to LDCT data collected for other purposes to identify patients at high risk of osteoporosis. In this study, about 68.3% of adults aged 50 years and older were estimated to have a low BMD. We calculated a prevalence of osteoporosis of 34.9% in women and 18.0% in men aged 50 years and older. The estimate for women is comparable to that based on DXA, but the prevalence in men was 2.4 times higher than that reported previously. It is important to note that this study does not aim to establish normative vBMD data for the general Korean population. Instead, it highlights the potential of using LDCT for opportunistic osteoporosis screening, particularly in specific patient populations, such as those undergoing lung cancer screening. Collecting data from a broader, more representative sample would be a rewarding follow-up study, requiring a different image dataset.

Worldwide, DXA remains the standard modality for diagnosing osteoporosis, but its inherent limitation of low sensitivity due to the 2D technique has been consistently noted [25]. In contrast, previous studies demonstrated that QCT may be more sensitive and accurate at diagnosing osteoporosis and predicting fracture risk than DXA, especially in men [26,27]. However, due to its complexity and labor- and time-intensive nature, the use of QCT in large-scale epidemiologic research studies is limited. The use of CT-based vertebral trabecular BMD assessment for opportunistic osteoporosis screening with automated approaches has garnered considerable attention in recent years. LDCT is frequently recommended for lung cancer screening and has been demonstrated to be associated with reduced lung cancer mortality [28]. A national lung cancer screening program based on LDCT was introduced in Korea in 2019. LDCT is suitable for opportunistic osteoporosis screening because both lung cancer and osteoporosis commonly affect individuals over 50 years of age, annual lung cancer screenings using LDCT are widely conducted, and LDCT scans generally include the upper lumbar vertebrae [29].

Our study primarily involved participants undergoing LDCT scans for lung cancer screening. While this cohort may not initially seem representative of the general population, it is important to note that in Korea, LDCT is commonly used not only for high-risk individuals but also increasingly in general health assessments. LDCT is recommended every two years for high-risk individuals aged 55–74. However, the national cancer screening program for those over 50 does not include LDCT for lung cancer, leading many Korean hospitals to operate their own health promotion centers that offer LDCT based on individual needs, regardless of smoking history. This approach broadens the demographic range of our study participants, making them more reflective of the general population. Nevertheless, it is important to clarify that our results should not be directly compared with BMD data from the general population without considering this specific screening context.

Large-scale epidemiologic studies have investigated the prevalence of osteoporosis in Korea using DXA data [4,30,31,32]. The “Osteoporosis and Osteoporotic Fractures Fact Sheet 2019” by the KSBMR analyzed DXA data from the KNHANES (2008–2011) and reported that 22.4% of Korean adults aged 50 and older have osteoporosis, and 47.9% have osteopenia. In other words, one in five Korean adults has osteoporosis and one in two has osteopenia. However, the osteoporosis prevalence rate was higher in the current study: 26.3% for osteoporosis and 39.0% for osteopenia in those aged 50 and older. Specifically, 34.9% of women over 50 were found to have osteoporosis, similar to the 37.3% diagnosed by DXA. In men over 50, osteoporosis prevalence was significantly higher when diagnosed by CT compared to DXA (18.0% vs. 7.5%) [4]. In a previous study in China conducted by Cheng et al., the prevalence of osteoporosis as assessed by QCT in men over 50 years was more than twice as high as that determined by DXA, consistent with our findings [33]. This discrepancy in osteoporosis prevalence may be due to variations in BMD measurement methods as well as racial differences from the international standard, which is based on data from Caucasian women. CT scans analyzed with advanced DL algorithms can detect subtle changes in bone density that DXA scans might overlook. This capability may lead to a higher detection rate of osteoporosis in populations, such as Korean men, where this condition has been underdiagnosed. Furthermore, our study cohort encompasses a broader spectrum of the male population, which historically has not been as thoroughly screened for osteoporosis as women. Our results may reflect a high incidence of spinal degeneration, aortic calcification, and vertebral fractures in elderly men, which can lead to underdiagnosis of low BMD by DXA [34].

Osteoporosis in men is a growing problem both worldwide and in Korea [35]. Although the prevalence of osteoporosis is higher in women, the mortality risk following a vertebral and non-vertebral fracture is higher and the treatment rate is lower in men [4]. Lim et al. reported that only 11% of 310 male patients aged 70 years or older had undergone a DXA scan, despite being required due to their age, with most participants being between 80 and 89 years old [36]. Similarly, Antonelli et al. reported that only 12.1% of women and 5.4% of men aged 65 and older with hip fractures underwent a DXA [37]. Additionally, Kiebzak et al. found that among 363 patients aged 50 and older with a history of non-traumatic hip fracture, only 27% of women and 11% of men had undergone a DXA within five years before fracture [38]. Furthermore, most clinical trials for osteoporosis treatment have primarily focused on postmenopausal women, meaning male osteoporosis is underestimated and overlooked. This disparity makes it challenging to manage osteoporosis in men effectively. However, there is little literature for vBMD from CT regarding this topic. Our findings underscore the necessity of increasing awareness and improving diagnostic and therapeutic strategies for osteoporosis in men to mitigate the associated fracture risks. There were sex differences in age-related changes in BMD. We observed a notable decrease in BMD values among women aged 50–54 years, but not among men. The sharper decrease in BMD among women aged 50 years and older is likely associated with the rapid reduction in estrogen production during menopause. This pattern appears consistent across female populations of diverse ethnicities [39].

DL offers substantial advantages for medical imaging applications, particularly in recognizing complex patterns within high-dimensional data, such as those from LDCT scans. DL excels at recognizing complex patterns in high-dimensional data, such as medical images from LDCT [40]. This capability is crucial for detecting subtle signs of conditions like osteoporosis, which may not be clearly visible with traditional imaging methods [41]. Unlike conventional machine learning techniques that require manual feature extraction, DL models autonomously learn the most predictive features directly from the data, enhancing accuracy while significantly reducing the preprocessing time and expertise needed. Additionally, DL methods can process large volumes of imaging data quickly and accurately, providing a significant advantage in clinical settings where timely diagnosis is critical. This operational efficiency supports the use of opportunistic screening during routine health checkups, improving the overall workflow and patient management across diverse medical imaging environments [42].

Our DL-based automatic BMD measurement software (ClariQCT Ver 1.0, ClariPi Inc., Seoul, Republic of Korea) can assess the trabecular BMD of the lumbar vertebral body using clinical CT scans without additional radiation exposure. Consequently, we were able to accurately measure CT-based BMD values in approximately 2000 subjects, stratified by age and sex. This study presents the first large-scale CT-derived BMD measurement dataset for Korean adults, though it is important to note that this dataset should not be considered a reference BMD dataset for the general Korean population. Instead, it provides valuable insights for specific patient populations, particularly those undergoing routine LDCT scans, such as lung cancer screenings.

The application of DL in analyzing LDCT scans offers a valuable tool for opportunistic osteoporosis screening during routine checkups, enabling the identification of individuals at risk without additional procedures. Our study shows a higher prevalence of osteoporosis in men when using CT-based methods compared to DXA, suggesting that traditional screening may underestimate this prevalence and highlighting the need for revised guidelines. The potential of DL-enhanced CT imaging to improve diagnostic accuracy and efficiency supports its broader use in healthcare and encourages further research and innovation in advanced imaging techniques. Future studies should aim to establish normative BMD data using a more representative sample of the general population.

However, there were several limitations to our study that should be considered. Firstly, validation studies in multicenter datasets and larger patient populations are needed. There may be regional bias in the data, as well as biases related to economic or disease status that influenced participants’ decisions to undergo a health checkup at a university hospital. Consequently, it is difficult to claim that the results are standards for the entire Korean population. Secondly, no DXA or QCT reference standards exist for the automated measurement of vBMD using DL. However, Oh et al. demonstrated that automated BMD measurement with DL allowed the accurate and reliable assessment of BMD, achieving AUC values of 0.943 and 0.990 for diagnosing osteoporosis and low BMD, respectively, using QCT as the reference standard in a clinical setting [19]. Thirdly, variations in diagnostic practices, examiner training, and equipment across the two centers could lead to differences in imaging quality and diagnostic outputs, potentially resulting in inter-inspector and inter-facility biases. To address this, we implemented rigorous preprocessing protocols and employed a sophisticated DL architecture that adapts to diverse imaging conditions. Despite these measures, the inherent differences in hardware among scanners present a limitation that must be considered when generalizing our findings across different clinical settings. Fourthly, we did not explore the relationship between BMD values and the risk of fragility, as fracture data were not available. Finally, the study subjects primarily consisted of individuals undergoing lung cancer screening, many of whom were likely smokers. As smoking is known to negatively influence bone health, a population predominantly composed of smokers may not accurately reflect the bone health of the general population. This could lead to an overestimation of osteoporosis prevalence when applying our findings more broadly [43].

## 5. Conclusions

In conclusion, DL-based opportunistic screening using LDCT scans can automatically measure BMD and offers a promising tool for estimating osteoporosis prevalence, identifying patients at high risk who may benefit from early intervention. However, it is important to acknowledge limitations such as variability in CT protocols and the need for further validation across diverse populations. Future research should focus on expanding the sample size and refining DL algorithms to establish this approach as a reliable clinical tool.

## Figures and Tables

**Table 1:** Low-dose chest CT parameters.

Center	*n*	CT Manufacturer	Tube Voltage	Slice Thickness
1	1157	Siemens (1108)	100 kVp (104)	3 mm
			120 kVp (1052)	
		Canon (49)		
			135 kVp (1)	
2	1007	GE (449)	120 kVp (1007)	0.668 mm (1)
				1.25 mm (1)
		Philips (520)		2.5 mm (895)
				3 mm (3)
		Canon (38)		4 mm (106)
				10 mm (1)

**Table 2:** Median and interquartile range (IQR) of volumetric bone mineral density values (mg/cm^3^) for each age group.

Age	*n*	Both	*n*	Female	*n*	Male
20~24	39	155.00 (141.00–176.00)	16	165.24 (153.67–177.72)	23	151.40 (132.49–167.10)
25~29	89	153.00 (138.00–170.50)	41	156.83 (139.40–181.70)	48	150.48 (135.41–165.52)
30~34	82	151.00 (132.00–169.00)	38	155.76 (139.50–187.07)	44	147.23 (128.27–167.42)
35~39	133	147.00 (125.50–161.50)	48	153.54 (138.55–171.79)	85	144.59 (122.23–158.32)
40~44	169	150.00 (127.00–170.50)	85	155.13 (133.80–173.66)	84	146.16 (123.29- 161.82)
45~49	171	141.00 (125.00–166.00)	86	150.37 (135.17–173.15)	85	133.47 (117.64- 159.32)
50~54	163	123.00 (105.00–146.00)	77	119.54 (101.83–141.69)	86	127.93 (107.57–147.40)
55~59	163	115.00 (92.00–138.00)	80	107.02 (87.47–126.80)	83	121.98 (96.35–143.29)
60~65	181	100.00 (85.00–116.50)	92	92.28 (80.43–112.78)	89	105.24 (91.68–126.81)
65~69	197	91.00 (72.50–117.00)	97	81.80 (62.52–103.53)	100	102.25 (82.98–127.53)
70~74	176	81.50 (61.25–110.00)	86	70.98 (53.83–92.41)	90	94.65 (74.89–129.30)
75~79	154	71.50 (55.75–94.00)	82	65.55 (43.70–81.64)	72	84.33 (64.70–104.40)
80~84	126	71.50 (48.50–101.25)	49	68.16 (40.80–92.54)	77	73.36 (55.59–105.58)
85~	72	69.50 (50.25–115.50)	31	60.07 (41.82–92.57)	41	86.08 (25.44–123.80)

**Table 3:** Age–standardized prevalence of osteoporosis and osteopenia in Korean adults aged ≥50 years, compared with published DXA data [4].

	Total		Men		Women	
Parameter	*n* (%)	Age-Standardized (%)	*n* (%)	Age-Standardized (%)	*n* (%)	Age-Standardized (%)
Current LDCT study	1915		1007		908	
≥50 years (*N*)	1232		638		594	
Osteoporosis	424 (34.4)	26.3	171 (26.8)	18.0	253 (42.6)	34.9
Osteopenia	480 (39.0)	42.0	254 (39.8)	42.4	226 (38.0)	41.5
Published DXA data						
≥50 years						
Osteoporosis	22.4		7.5		37.3	
Osteopenia	47.9		46.8		48.9	

## Data Availability

Data supporting the present study are available from the corresponding author upon reasonable request.
